# PSMD2 contributes to the progression of esophageal squamous cell carcinoma by repressing autophagy

**DOI:** 10.1186/s13578-023-01016-4

**Published:** 2023-03-30

**Authors:** Yachen Liu, Meng Wu, Shuxiang Xu, Xiangjie Niu, Weiling Liu, Chuanwang Miao, Ai Lin, Yang Xu, Lili Yu

**Affiliations:** 1 Department of Cardiology, Cardiovascular Key Lab of Zhejiang Province, The Second Affiliated Hospital, School of Medicine, Zhejiang University, Hangzhou, 310009 China; 2 Department of Medical Oncology, Key Laboratory of Cancer Prevention and Intervention, Ministry of Education, The Second Affiliated Hospital, School of Medicine, Zhejiang University, Hangzhou, 310009 China; 3 Department of Thoracic Surgery, Fudan University Shanghai Cancer Center, Shanghai, China; 4 Department of Oncology, Shanghai Medical College, Fudan University, Shanghai, China; 5 Department of Etiology and Carcinogenesis, Chinese Academy of Medical Sciences and Peking Union Medical College, Beijing, 100021 People’s Republic of China

**Keywords:** PSMD2, Autophagy, Proliferation, Proteomics, ASS1, Esophageal squamous cell carcinoma

## Abstract

**Background:**

The ubiquitin–proteasome and autophagy-lysosomal systems collaborate in regulating the levels of intracellular proteins. Dysregulation of protein homeostasis is a central feature of malignancy. The gene encoding 26S proteasome non-ATPase regulatory subunit 2 (*PSMD2*) of the ubiquitin–proteasome system is an oncogene in various types of cancer. However, the detailed role of PSMD2 in autophagy and its relationship to tumorigenesis in esophageal squamous cell carcinoma (ESCC) remain unknown. In the present study, we have investigated the tumor-promoting roles of PSMD2 in the context of autophagy in ESCC.

**Methods:**

Molecular approaches including DAPgreen staining, 5-Ethynyl-2ʹ-deoxyuridine (EdU), cell counting kit 8 (CCK8), colony formation, transwell assays, and cell transfection, xenograft model, immunoblotting and Immunohistochemical analysis were used to investigate the roles of PSMD2 in ESCC cells. Data-independent acquisition (DIA) quantification proteomics analysis and rescue experiments were used to study the roles of PSMD2 in ESCC cells.

**Results:**

We demonstrate that the overexpression of PSMD2 promotes ESCC cell growth by inhibiting autophagy and is correlated with tumor progression and poor prognosis of ESCC patients. DIA quantification proteomics analysis shows a significant positive correlation between argininosuccinate synthase 1 (ASS1) and PSMD2 levels in ESCC tumors. Further studies indicate that PSMD2 activates the mTOR pathway by upregulating ASS1 to inhibit autophagy.

**Conclusions:**

PSMD2 plays an important role in repressing autophagy in ESCC, and represents a promising biomarker to predict prognosis and a therapeutic target of ESCC patients.

**Supplementary Information:**

The online version contains supplementary material available at 10.1186/s13578-023-01016-4.

## Background

Human cells have evolved two major systems, the ubiquitin–proteasome system (UPS) and autophagy, to maintain their protein homeostasis by degrading proteins and organelles. The UPS mostly degrades unfolded single polypeptides whereas autophagy primarily deals with larger cytosolic structures. Proteasome- and autophagy-mediated protein degradation plays coordinated and complementary roles in maintaining protein quality control [1–3]. Cancer can develop when the homeostasis of different proteins is broken, and therefore, is often characterized by the accumulation of mutant or dysregulated proteins in malignant cells. The mammalian target of rapamycin complex 1 (mTORC1) is a key regulator that senses intracellular protein quality and quantity. In the presence of sufficient levels of amino acids, mTORC1 is activated to promote anabolic processes; however, upon the depletion of free amino acids, mTORC1 is inactivated, triggering the switch to the autophagy-inducing catabolic state [4–6].

Many UPS components directly promote or suppress tumorigenesis, depending on the context and cancer types [7]. For instance, PSMD4, a non-ATPase subunit of the 19S regulatory particle (RP) of the proteasome, has been identified as an oncoprotein in gastrointestinal stromal tumors and melanoma [8, 9]. Recently, PSMD2 has been characterized as a ubiquitin receptor in the 19S RP of the 26S proteasome, which is responsible for substrate recognition and binding [10, 11]. A previous study has reported that knockdown of PSMD4 activates the autophagic compensatory pathway [12], suggesting that the PSMD family might also be involved in autophagy. It has been shown that overexpression of PSMD2 positively correlates with cell proliferation, invasion and metastasis, and poor prognosis in many types of primary tumors [13–16]. However, the underlying mechanism is largely unknown. Whether PSMD2 plays a role in autophagy remains an interesting research subject.

Esophageal squamous cell carcinoma (ESCC) is one of the most common malignancies and the sixth leading cause of cancer-related death worldwide [17]. With a high metastatic potential, the prognosis of this malignancy is relatively poor [18–21]. ESCC lacks effective targeted therapies, mainly because of the lack of the mechanistic understanding of this disease and the potential molecular targets for its treatment. Thus, a better understanding of the molecular mechanisms implicated in ESCC progression may lead to the identification of new diagnostic approaches and prognostic markers. Recently, we have performed whole-genome and transcriptome sequencing of ESCC [22], and by deeply mining the data, we have found that PSMD2 overexpression is associated with poor survival of the ESCC patients, suggesting that PSMD2 is an important molecule in the development of ESCC. In the present study, we aimed to investigate the roles of PSMD2 in ESCC, providing novel insights into ESCC pathogenesis.

## Methods

### Clinical samples and study subjects

Primary ESCC specimens and their matched adjacent nontumor tissues (≥ 5 cm from tumor site) were obtained with written informed consent from 144 ESCC patients who underwent esophagectomy without chemotherapy or radiotherapy before surgery at Linzhou Cancer Hospital (Linzhou, Henan, China) between September 2015 and September 2016.

### Cell lines and culture

ESCC cell lines (KYSE30 and KYSE450) were kind gifts from Dr. Y. Shimada of Hyogo College of Medicine (Nishinomiya, Hyogo, Japan). All ESCC cell lines were maintained in RPMI 1640 medium (Corning, Manassas, VA, USA) supplemented with 10% fetal bovine serum (FBS, HyClone, Logan, Utah, USA) at 37 °C in a humidified incubator with 5% CO2. All cell lines were authenticated by DNA fingerprinting analysis and tested for mycoplasma contamination.

### Lentiviral production and infection

Tumor cells were infected with either pLKO.1-U6-T2A-puro lentiviral vectors (Hanbio, Shanghai, China) containing short hairpin RNA (shRNA) sequences against either luciferase (as a nontarget control) or human *PSMD2* (IGEbio, Guangzhou, Guangdong, China). KYSE30 and KYSE450 cells were infected with the virus and cultured in a complete medium for 24 h. Infected cells were selected with 2 μg/mL puromycin (MCE, Beijing, China). For the stable overexpression of *PSMD2*, pHBLV-CMV-MCV-MCS-EF1-ZsGreen-T2A-puro (Hanbio, Shanghai, China) lentiviral vector was used.

### Transient overexpression and RNA interference

*The plasmids for PSMD2* (#23,850–1) and *ASS1* (GV362) transient overexpression were purchased from (GeneChem, Shanghai, China). Plasmid transfections were performed using Lipofectamine 3000 (Invitrogen, Carlsbad, CA, USA). Small interfering RNAs (siRNAs) targeting *PSMD2*, *ASS1,* or *ATG7* (JTS, Beijing, China) were transfected into cells with Lipofectamine RNAiMAX Reagent (Invitrogen, Carlsbad, CA, USA). The sequences of siRNAs are shown in Additional file 1: Table S1.

### Western blotting analysis

Cells transfected with siRNA, plasmid, lentivirus were harvested in sample buffer supplemented with Protease/Phosphatase Inhibitor Cocktail (#5872, Cell signaling Technology, Shanghai, China). Protein concentrations were detected by bicinchoninic acid (BCA) assay (P0010S, Beyotime, Shanghai, China) according to the manufacturer’s instructions. The antibodies against PSMD2 (ab197054, 1:1000 dilution), GAPDH (ab181602, 1:10000 dilution), Atg7 (ab52472, 1:100000 dilution) were purchased from Abcam (Cambridge, UK). The antibodies against mTOR (2972, 1:1000 dilution), phospho-mTOR (2971, 1:1000 dilution), p62 (8025, 1:1000 dilution), Beclin-1 (3495, 1:1000 dilution) or LC3A/B (12741, 1;1000 dilution) were from Cell Signaling Technology (Beverly, Massachusetts, USA). The antibody against ASS1(16210–1-AP, 1;1000 dilution) was purchased from Proteintech (Beijing, China).

### Cell proliferation assays

In vitro cell proliferation was examined by cell growth, EdU (Beyotime Biotechnology, Shanghai, China) incorporation, and colony formation assays. For cell growth assays, cells were seeded in 96-well plates at a density of 2500–3500 cells/well in 100 μL of cell suspension. After a certain time in culture, cell viability was measured with the CCK-8 kit (Dojindo, Kumamoto, Japan) at 24, 48, 72, and 96 h. Each assay with 6 replicates was repeated 3 times. For EdU incorporation assays, 5 × 10^5^ cells were plated on coverslips in 6-well culture plates containing RPMI 1640 medium supplemented with 10% FBS and then analyzed according to the manufacturer’s instructions. Images were captured by confocal microscopy, and the percentage of EdU-positive cells calculated using the formula EdU-positive cell count/total cell count × 100%. Each assay was performed in triplicate. For colony formation assays, cells were plated into 6-well culture plates at 1000–3000 cells/well, cultured for 14 days before being fixed with 4% paraformaldehyde (Sangon Biotech, Shanghai, China), and stained with 0.5% crystal violet (C0121, Beyotime, Shanghai, China).

### Invasion and migration assays

The invasion assays were performed in 24-well Millicell chambers in triplicate. The 8-μm pore inserts were coated with 60 µL of Matrigel (BD Biosciences, Franklin Lakes, New Jersey, USA). Cells (5 × 10^4^ per well) were added to coated filters in 150 µL of serum-free medium. The medium containing 20% FBS was added to the lower chamber as a chemoattractant. After 16–18 h at 37 °C in a 5% CO_2_ incubator, the matrigel coating on the upper surface of the filter was removed. Cells that migrated through the filters were fixed with 4% methanol (Sangon Biotech, Shanghai, China), stained with 0.5% crystal violet, and photographed with a microscope (Olympus, Shanghai, China). The number of cells in 3 random fields was counted. The migration assay was conducted similarly without coating with matrigel.

### Xenograft mouse models

ESCC cells (1 × 10^7^ KYSE30 cells, or 5 × 10^6^ KYSE450 cells) with the overexpression or silence of PSMD2 were subcutaneously injected into the armpit of 4-week-old female NOD/*scid* mice (5–8 mice per group) purchased from the Beijing Huafukang bioscience company in China, maintained in animal facility of a controlled condition (23 ± 1 °C, 50 ± 10% humidity and 12–12 h light–dark cycle). Tumor volume was measured 3 times a week for 4 weeks and calculated using the formula Volume = 0.5 × Width^2^ × Length. When the tumors had reached a volume of approximately 1500 mm^3^, the mice were euthanized by carbon dioxide inhalation, the tumors were excised and embedded in paraffin for IHC analysis. For the treatment part, one week after KYSE450 cells transplantation, mice were randomly divided into 2 groups and treated with Everolimus (RAD001) (MCE, Beijing, China) intragastrically (5 mg/kg) every other day for 14 days. Tumors were measured 3 times a week and volume was counted.

### Autophagy detection

A total of 3 × 10^5^ ESCC cells were seeded in glass-bottom cell culture dishes. Autophagy was induced by culturing in serum-free medium for 3 h or 20 h. After the supernatant was discarded, the cells were incubated with DAPGreen (Dojindo, Kumamoto, Japan) working solution for 30 min at 37 °C. After washing with serum-free medium twice, cell nuclei were stained with 1 μg/ml Hoechst 33342 (Thermo, Rockford, Illinois, USA) and examined under a confocal fluorescence microscope (Leica, Wetzlar, Germany). Images in the same panel were taken under the same excitation conditions to precisely examine autophagy under different conditions. Autophagy was also examined with the ratio of LC3B-II to LC3B-I by immunoblot analysis. The protein bands were quantified by gray scanning. Cells were also treated with 80 μM chloroquine diphosphate (CQ, MCE) for 2 h to inhibit lysosome function. The mRFP-GFP-LC3 lentiviral was obtained from GeneChem (Shanghai, China). 10^4^ ESCC cells were plated in 24-well plates, followed by incubation in 1640 with lentiviral for 24 h. LC3 puncta were detected with Zeiss LSM980 confocal microscope fitted with a × 63 oil immersion objective.

### Sample preparation and DIA

Total cellular protein extracted from *PSMD2* knockdown cells and shControl cells was examined with data-independent acquisition (DIA) proteomics by BGI Genomics (Shenzhen, Guangdong, China). Differential expression analysis was used to identify the potential downregulated proteins after *PSMD2* knockdown.

### Immunohistochemical analysis

Paraffin-embedded tissue section and microarray were incubated with antibodies against PSMD2 (1:200; ab197054, Abcam, Cambridge, UK), ASS1 (1:200; 16210–1-AP, Proteintech, Beijing, China), LC3 (1:200; 14600–1-AP, Proteintech, Beijing, China) and p62 (1: 200; 88588, Cell Signaling.

Technology, Massachusetts, USA) at 4 °C overnight, and detected with the ABC Kit (Pierce, Rockford, Illinois, USA). The labeling score of intensity was estimated as negative (0), weak (1), moderate (2), or strong (3). The level of staining, defined as the percentage of positively stained cells in all cells, was scored as 1 (≤ 10%), 2 (11–50%), 3 (51–80%), or 4 (> 80%). The total immunoreactive score was obtained by multiplying the intensity score with the levels of staining, ranging from 0 to 12.

### Statistical analysis

We used the Student’s t-test when samples were not paired and the paired t-test for paired samples. When more than two variables were compared, either one-way ANOVA or Kruskal–Wallis test was used. The Fisher exact test was used in the analysis of contingency tables. Spearman’s rank correlation coefficient was used to measure the correlation between two continuous variables. We used the log-rank test in univariate survival analysis and the Cox proportional hazards model in multivariate survival analysis. The Kaplan–Meier plot was used for presentation. All statistical tests were two-tailed and *P* < 0.05 was considered significant. Analyses were performed using Prism 6.0 (Graphpad Software Inc).

## Results

### PSMD2 inhibits autophagy and promotes the proliferation of ESCC cells

**Fig. 1:**
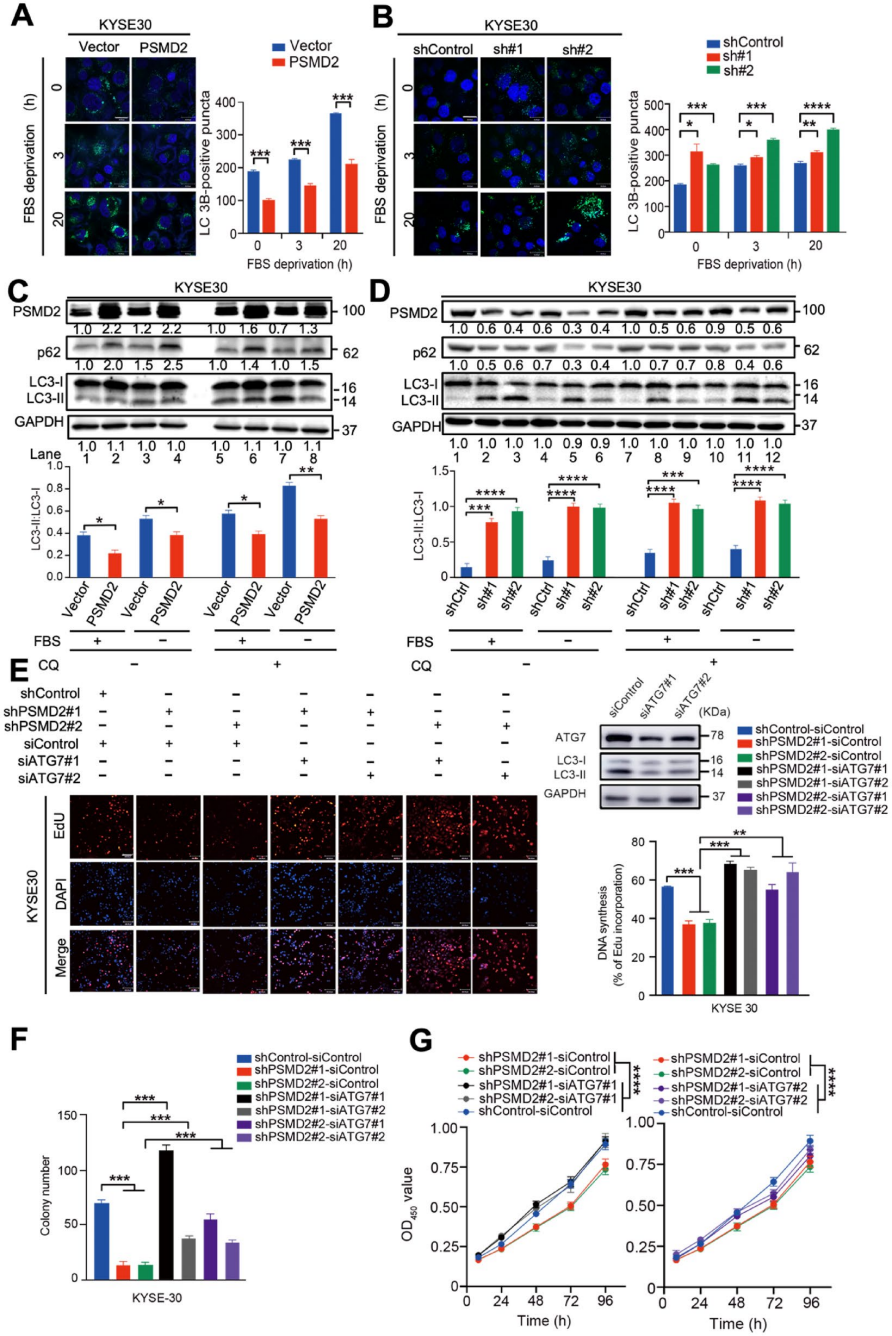
PSMD2 inhibits autophagy and promotes the proliferation of KYSE30. **A** The overexpression of *PSMD2* reduced the formation of LC3B puncta. **B** The knockdown of *PSMD2* increased the formation of LC3B puncta. Autophagy was induced in KYSE30 cells with *PSMD2* overexpression or knockdown by FBS deprivation for 3 h or 20 h. The cells were then stained with the DAPGreen (green) with nucleus counterstained with Hoechst 33342 (blue). Scale bar: 30 μm. **C**, **D** PSMD2 inhibited autophagy under FBS deprivation. KYSE30 cells with *PSMD2* overexpression (PSMD2) (**C**) or *PSMD2* knockdown (shPSMD2) (**D**) were treated with CQ as indicated. The cell lysate was examined by immunoblotting to detect p62, LC3 and GAPDH. The LC3-II: LC3-I ratio was qualified with ImageJ software. **E** Effect of *ATG7* knockdown on the proliferation of ESCC cells with *PSMD2* depletion was examined by fluorescently staining with EdU (red). The nucleus was stained with DAPI (blue). The efficiency of *ATG7* knockdown was shown in the upper right panel. The percentage of EdU-positive cells was shown in the bottom right panel. Scale bar: 100 μm. **F** Effect of *ATG7* knockdown on colony formation of ESCC cells with *PSMD2* depletion. N = 3. The data were presented as mean ± SD. **G** Effect of *ATG7* knockdown in ESCC cells with *PSMD2* depletion on cell proliferation. The cells were used to determine the cell proliferation rate by CCK-8 assay at the indicated time point. N = 3. The data were presented as mean ± SD, * for *p* < 0.05, ** for *p* < 0.01, *** for *p* < 0.001 and **** for *p* < 0.0001

Since PSMD2 is a component of UPS and UPS impairment may activate selective autophagy [23, 24], we hypothesized that PSMD2 itself may be directly involved in autophagy. To test this notion, we treated ESCC cells after fetal bovine serum (FBS) deprivation, which has been shown to induce autophagy [25, 26]. We found that, in response to FBS deprivation, the puncta of microtubule-associated protein light chain 3 (LC3), a marker protein involved in the formation of autophagosomes, were significantly decreased in cells with *PSMD2* overexpression but significantly increased in cells with *PSMD2* knockdown as compared with control cells without the *PSMD2* expression change (Fig. 1A, B, Additional file 1: Fig. S1A, B). We also examined the autophagy with the autophagy flux. KYSE30 and KYSE450 cells expressing tandem mRFP-GFP-tagged LC3B were treated with FBS deprivation for 20 h. We observed a significant increase of RFP + GFP + signal (autophagosome) in *PSMD2* knockdown ESCC cells (Additional file 1: Fig. S2A, B). Suggesting that PSMD2 plays an important role in autophagy. When treated with or without the lysosomal inhibitor chloroquine diphosphate (CQ) [27, 28], p62, a receptor for autophagy, were significantly increased in ESCC cells with *PSMD2* overexpression and decreased in cells with *PSMD2* knockdown when compared with control cells (Fig. 1C, D). The ratios of LC3-II to LC3-I were significantly lower in ESCC cells with *PSMD2* overexpression but higher in cells with *PSMD2* knockdown when compared with control cells (Fig. 1C, D). These results indicate that PSMD2 inhibits autophagosome formation in ESCC cells after FBS deprivation independently of the lysosome function. To examine if PSMD2-mediated autophagy is involved in the malignant phenotype of ESCC cells, we abolished the autophagic activity in *PSMD2*-silenced ESCC cells by depleting autophagy-related 7 (ATG7) [29]. Our results showed that the depletion of ATG7 expression could abrogate the inhibition of ESCC cell proliferation and colony formation caused by *PSMD2* silencing (Fig. 1E–G, Additional file 1: Fig. 1C–E). Together, these results suggest that PSMD2 could promote the growth of ESCC cells by inhibiting autophagy.

### PSMD2 promotes the progression of ESCC

**Fig. 2:**
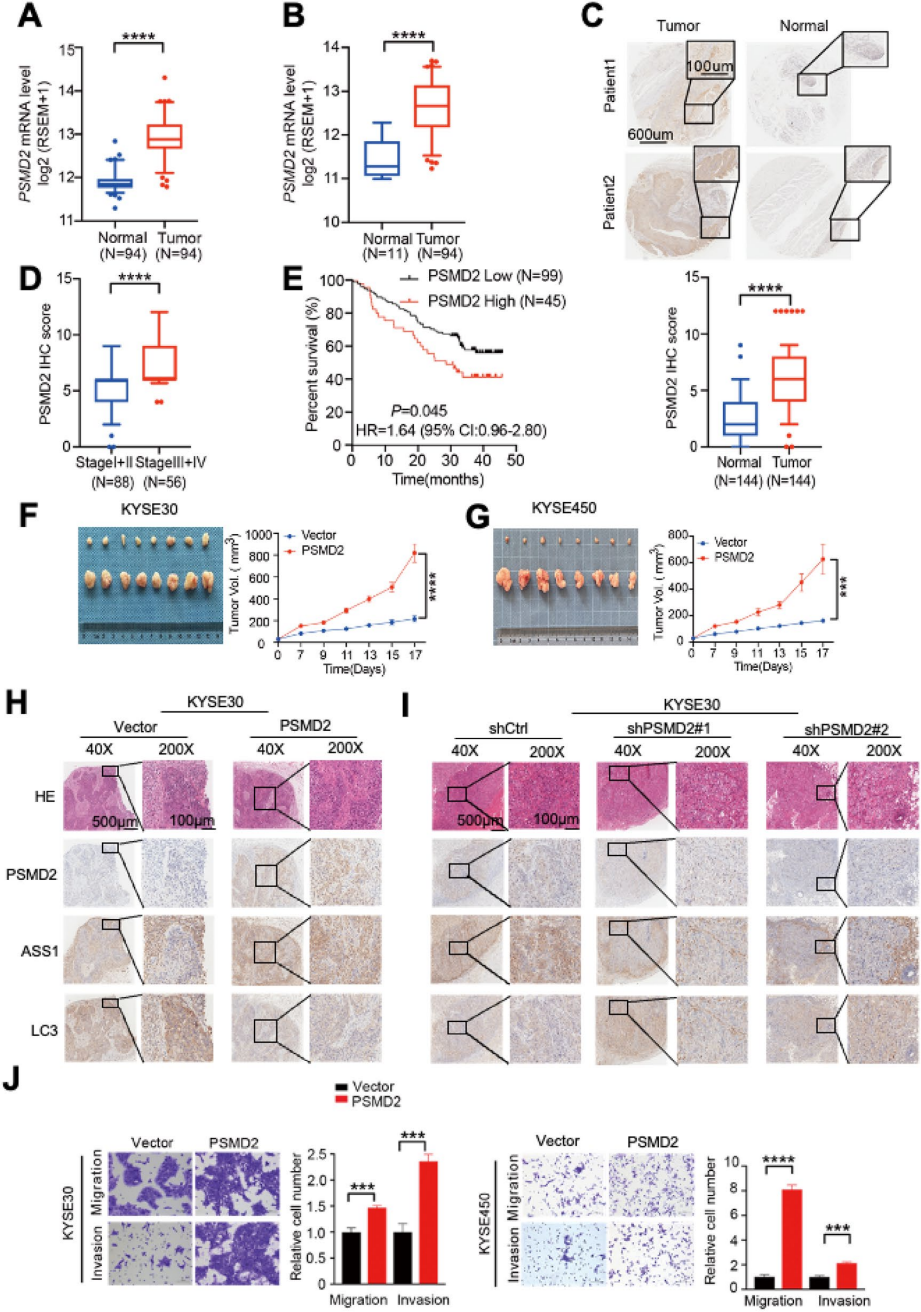
PSMD2 promotes the proliferation of ESCC. **A** The expression levels of *PSMD2* mRNA in ESCC samples and normal controls. Sample sets were previously described [22]. **B** The expression levels of *PSMD2* mRNA in ESCC samples and normal controls of the esophageal cancer dataset (ESCA) from TCGA database. **C** IHC staining of PSMD2 in tissue microarray consisting of 144 ESCC samples. Upper panel, representative IHC images, Scale bar: 600 μm (left images) and 100 μm (right images); lower panel, statistical analysis of the data. **D** Box plots of tissue array showing PSMD2 expression levels in early and advanced stages of ESCC. **E** Kaplan–Meier estimates of survival time of 144 ESCC patients by different PSMD2 expression levels in tumor tissues. The best-performing threshold was used as a cutoff. **F**, **G** Representative images of the xenograft tumors formed in NOD/*scid* mice inoculated with *PSMD2* overexpressing KYSE30 cells (**F**) and KYSE450 cells (**G**). **H-I** Representative H&E and IHC images show the expression of PSMD2, ASS1, LC3 in tumors formed by KYSE30 cells with of *PSMD2* overexpression (**H**) or *PSMD2* knockdown (**I**). **J** *PSMD2* overexpression significantly promoted the migration and invasion capabilities of ESCC cells. The left panels were representative images of ESCC cells in transwell assays and the right panels represent statistical analysis. N = 3. Data were presented as mean ± SD, ***p < 0.001, and ****p < 0.0001

We then analyzed the *PSMD2* mRNA expression levels in ESCC and paired adjacent normal tissues from 94 individuals [22] and found that the *PSMD2* mRNA expression levels were significantly higher in tumors than in normal tissues (Fig. 2A). Analysis of the TCGA esophageal cancer dataset (ESCA) also revealed significantly higher *PSMD2* expression in tumors than in normal tissues (Fig. 2B). We found that *PSMD2* was significantly overexpressed in ESCC when compared to the normal tissues (fold change = 2.05, FDR = 4.58e-32; Additional file 1: Table S2). Immunohistochemical (IHC) staining of tissue arrays (*n* = 144) showed that 73.6% (106/144) of ESCC had higher PSMD2 protein levels than their adjacent normal tissues (*p* < 0.0001; Fig. 2C). The higher PSMD2 levels were more evident in advanced ESCC (III and IV stages) compared to those of early I and II stages (*p* < 0.0001; Fig. 2D), and were correlated with the poor prognosis of ESCC patients (*p* = 0.045 for the log-rank test; Fig. 2E). Mouse xenograft experiments showed that tumors formed by *PSMD2* overexpressing ESCC cells grew significantly faster than tumors formed by control ESCC cells (Fig. 2F, G). Consistent with this finding, the growth of tumors formed by *PSMD2* silenced ESCC cells were dramatically suppressed compared with that of tumors formed by control ESCC cells (Additional file 1: Fig. S3A, B). We also examined the relationship between the PSMD2, ASS1, LC3 and p62 expression levels determined by IHC staining in mice xenografts derived from ESCC cells with the *PSMD2* expression changes, and the results were consistent with the in vitro findings that PSMD2 increases the expression of ASS1 and inhibits autophagy (Fig. 2H, I, Additional file 1: Fig. S4A–D). In addition, *PSMD2* overexpression significantly promoted the migration and invasion capabilities of ESCC cells, and *PSMD2* knockdown inhibited the migration and invasion capabilities of ESCC cells (Fig. 2J, Additional file 1: Fig. S3C). Together, these results support the notion that PSMD2 promotes ESCC progression.

### PSMD2-ASS1 pathway promotes ESCC proliferation by inhibiting autophagy

**Fig. 3:**
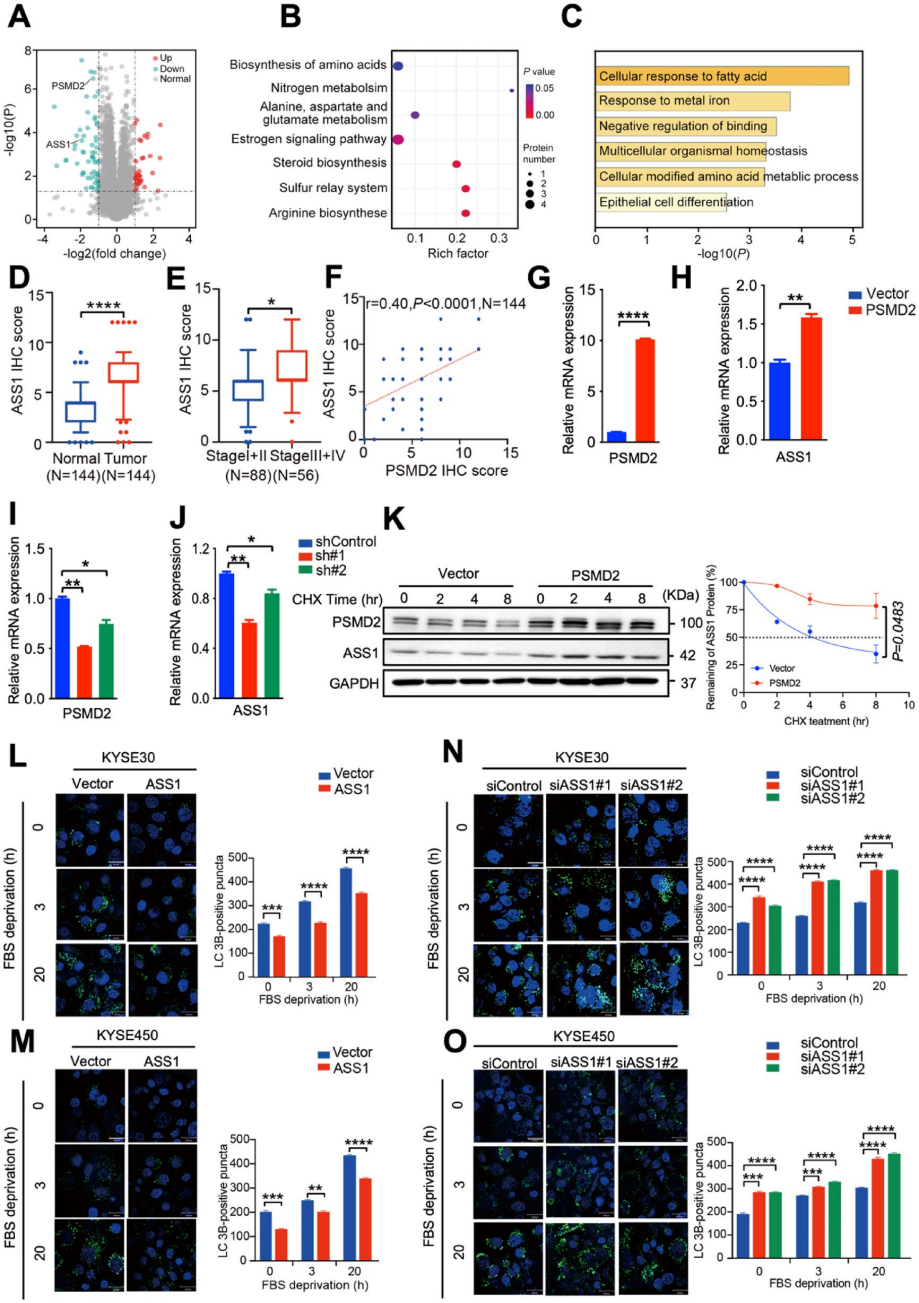
PSMD2 inhibits autophagy by inducing ASS1. **A** Differential expression analysis of proteomics data of KYSE30 cells with or without *PSMD2* knockdown. **B** Functional enrichment of the differentially expressed proteins in KYSE30 cells with or without *PSMD2* knockdown by KEGG. **C** Functional enrichment of the differentially expressed proteins in KYSE30 cells with or without *PSMD2* knockdown by Metascape. **D** Statistical analysis of the protein levels of of ASS1 detected by IHC of tissue microarray consisting of 144 ESCC and normal samples. **E** Box plots showing ASS1 expression levels in the early and advanced stage of ESCC. Data were derived from tissue microarray. **F** The positive correlation between the protein levels of PSMD2 and ASS1 proteins in ESCC tissues. N = 144. **G-H** KYSE450 cells with *PSMD2* overexpression were examined for *PSMD2* mRNA levels (**G**) and *ASS1* mRNA levels (**H**) by qPCR.**I**, **J** KYSE450 cells with *PSMD2* knockdown were examined for *PSMD2* mRNA levels (**I**) and *ASS1* mRNA levels (**J**) by qPCR.**K** KYSE30 cells with *PSMD2* overexpression or vector were treated with CHX (100 μg/ml). The protein level of ASS1 was examined at 0, 2, 4, 8 h by immunoblotting (left panel) and protein half-life calculated (right panel).**L**, **M** *ASS1* overexpression reduced the formation of LC3B puncta. Autophagy was induced in KYSE30 cells (**L**) and KYSE450 cells (**M**) with *ASS1* overexpression by FBS deprivation. The cells were then stained with the DAPGreen (green). The nucleus was stained with Hoechst 33342 (blue). Scale bar: 30 μm. Quantitative analysis of the autophagic puncta was shown in the right panel. N = 3. Data were presented as mean ± SD. **N–O** *ASS1* knockdown in KYSE30 cells (N) and KYSE450 cells (**O**) increased the formation of LC3B puncta. N = 3. Data were presented as mean ± SD

To elucidate the molecular mechanism underlying the inhibitory effect of PSMD2 on autophagy, we used data-independent acquisition quantification proteomics analysis to profile the differentially expressed proteins in KYSE30 cells with or without *PSMD2* silencing. Protein candidates were divided into up- or down-regulated groups (Fig. 3A), identifying 62 downregulated and 34 upregulated proteins (Additional file 1: Table S3). Both KEGG (Kyoto Encyclopedia of Genes and Genomes) and Metascape analysis revealed that the differentially expressed proteins in cells after *PSMD2* silencing were enriched in the the arginine biosynthesis pathway with the expression levels of *ASS1* gene encoding argininosuccinate synthase 1 about twofold downregulated in cells with *PSMD2* silencing compared with that in control cells (Fig. 3B, D). In addition, the expression levels of ASS1 levels in ESCC samples were significantly higher than those in adjacent normal tissues (p < 0.0001; Fig. 3D), and the higher expression levels of ASS1 were correlated with advanced clinical stages (p = 0.0134; Fig. 3E). The expression levels of ASS1 in ESCC tumors were positively correlated with PSMD2 levels, further suggesting that the ASS1 expression may be regulated by PSMD2 (Fig. 3F, Additional file 1: Fig. S5A). We also showed that *PSMD2* induces the levels of *ASS1* mRNA (Fig. 3G–J). In support of this notion, PSMD2 increased the stability of ASS1 protein in ESCC cells (Fig. 3K). ASS1 had the same function on autophagosome formation and proliferation of ESCC cells as PSMD2, suggesting that the tumorigenic roles of PSMD2 in ESCC progression are mediated by ASS1 (Fig. 3L–O, Additional file 1: Fig. S5B, C).

**Fig. 4:**
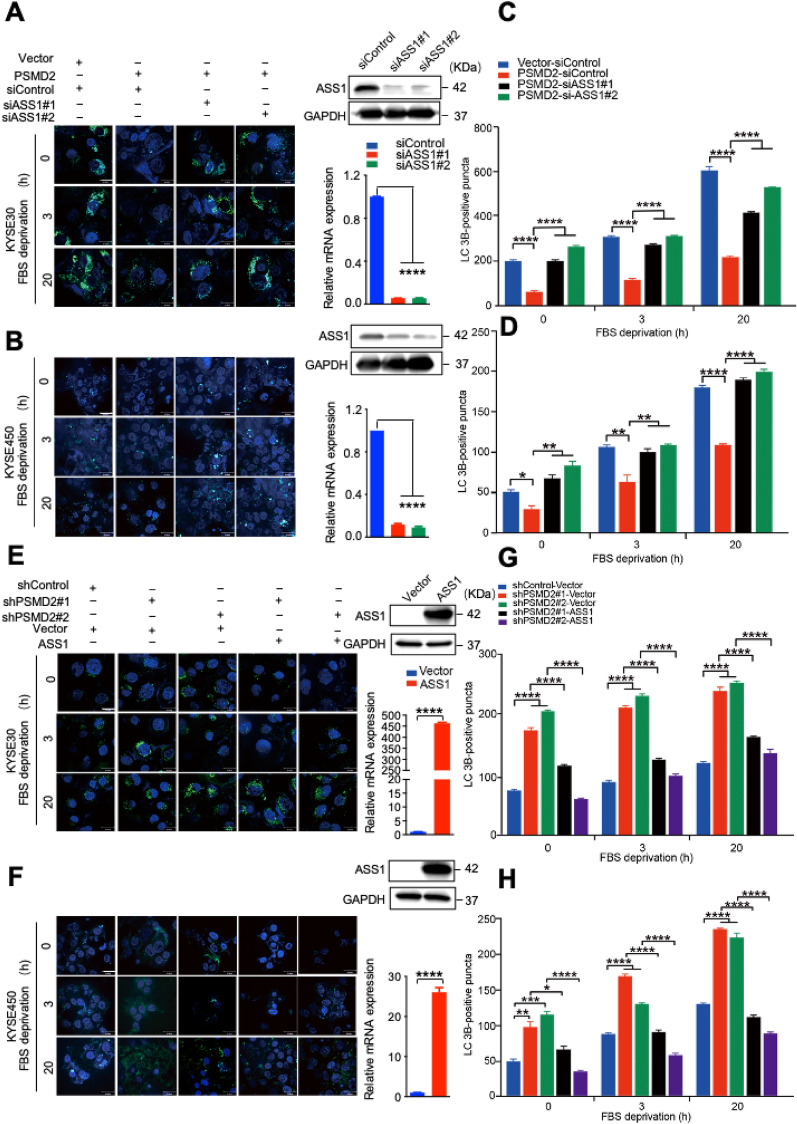
The involvement of ASS1 in PSMD2-mediated inhibition of autophagy. **A–D** The impact of *ASS1* knockdown on the formation of LC3B puncta in KYSE30 cells (**A**) and KYSE450 cells (**B**) with *PSMD2* overexpression. The efficiency of *ASS1* knockdown was shown in the right panel. Protein levels, upper right panel. RNA levels, bottom right panel. Autophagy was induced by FBS deprivation then stained with the DAPGreen (green). The nucleus was stained with Hoechst 33342 (blue). Scale bar: 30 μm. Quantitative analysis of the autophagic puncta of KYSE30 cells (**C**) and KYSE450 cells (**D**) was shown. N = 3.**E–H** The impact of *ASS1* overexpression on the formation of LC3B puncta in KYSE30 cells (**E**) and KYSE450 cells (**F**) with *PSMD2* knockdown. The treatment conditions were the same as those in (**A**, **B**). The efficiency of *ASS1* overexpression was shown in the right panel. Protein levels, upper right panel. RNA levels, bottom right panel. Quantitative analysis of the autophagic puncta of KYSE30 cells (**G**) and KYSE450 cells (**H**) was shown. N = 3. Data were presented as mean ± SD, *p < 0.05, **p < 0.01, and****p < 0.0001

To determine the involvement of ASS1 in mediating roles of PSMD2 in autophagy, we silenced *ASS1* expression in cells with *PSMD2* overexpression or overexpressed *ASS1* in cells with *PSMD2* depletion, and examined their autophagy state. The results showed that silencing *ASS1* expression in cells with *PSMD2* overexpression reversed the phenotype of decreased autophagosome formation in PSMD2 expressing cells (Fig. 4A–D). In addition, the overexpression of *ASS1* in cells with *PSMD2* depletion reversed the phenotype of increased autophagosome formation in *PSMD2* silenced cells (Fig. 4E–H). Therefore, ASS1 plays an important role in mediating PSMD2-dependent inhibition of autophagy.

**Fig. 5:**
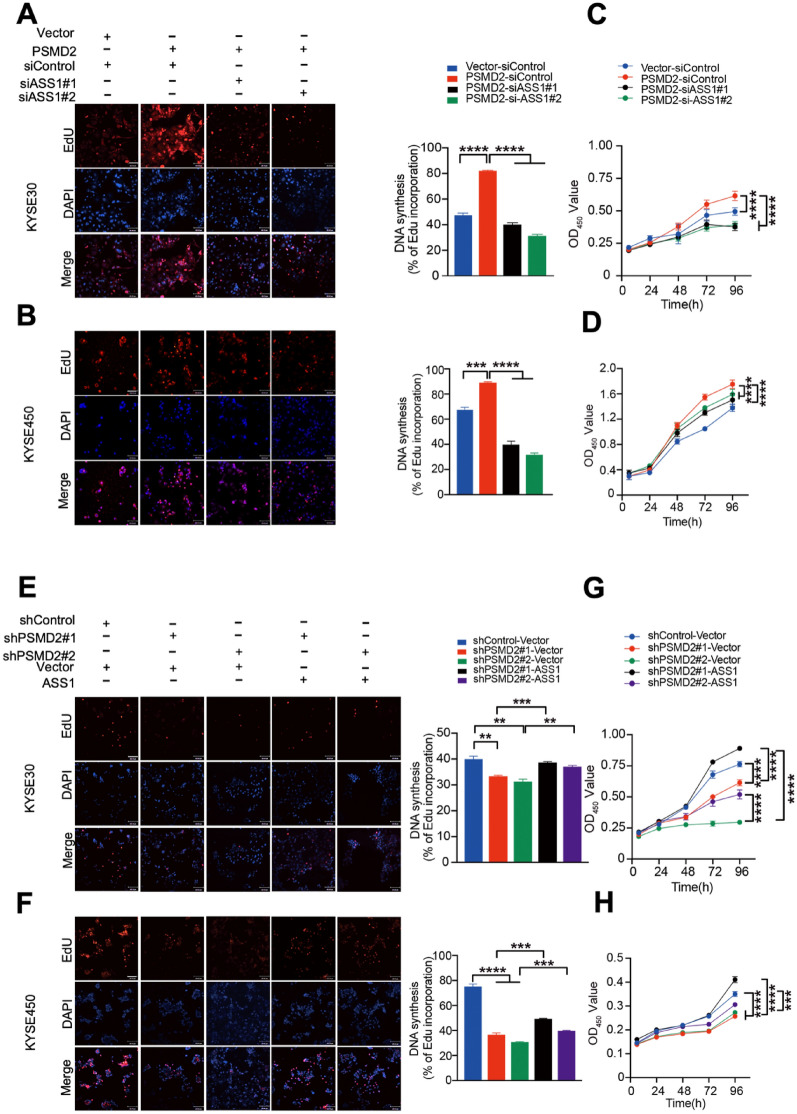
The involvement of ASS1 in the proliferation promoted by PSMD2. **A**, **B** The impact of *ASS1* knockdown on the proliferation of KYSE30 cells (**A**) and KYSE450 cells (**B**) with *PSMD2* overexpression. The cells were fluorescently stained with EdU (red). The nucleus was stained with DAPI (blue). The percentage of EdU-positive cells was calculated and shown in the right panel. Scale bar: 100 μm. **C**, **D** The impact of *ASS1* knockdown on the proliferation of KYSE30 cells (**C**) and KYSE450 cells (D) with *PSMD2* overexpression. **E**, **F** The impact of *ASS1* overexpression on the proliferation of KYSE30 cells (**E**) and KYSE450 cells (**F**) with *PSMD2* knockdown. The cells were fluorescently stained with EdU (red). The nucleus was stained with DAPI (blue). The percentage of EdU-positive cells was shown in the right panel. N = 3. **G-H** The impact of *ASS1* overexpression on the proliferation of KYSE30 cells (**G**) and KYSE450 cells (**H**) with *PSMD2* depletion. N = 3. Data were presented as mean ± SD, ***p < 0.001, and****p < 0.0001

Consistent with the conclusion that ASS1 mediates the PSMD2-dependent inhibition of autophagy, we found that silencing *ASS1* expression in cells with *PSMD2* overexpression reversed the phenotype of increased proliferation and colony formation of ESCC cells (Fig. 5A–D Additional file 1: Fig. S6A). In addition, the overexpression of *ASS1* in cells with *PSMD2* depletion reversed the phenotype of decreased proliferation and colony formation of ESCC cells (Fig. 5E–H, Additional file 1: Fig. S6B). These results indicate that PSMD2-ASS1 pathway promotes ESCC proliferation by inhibiting autophagy.

### PSMD2 inhibits autophagy by activating mTOR

**Fig. 6:**
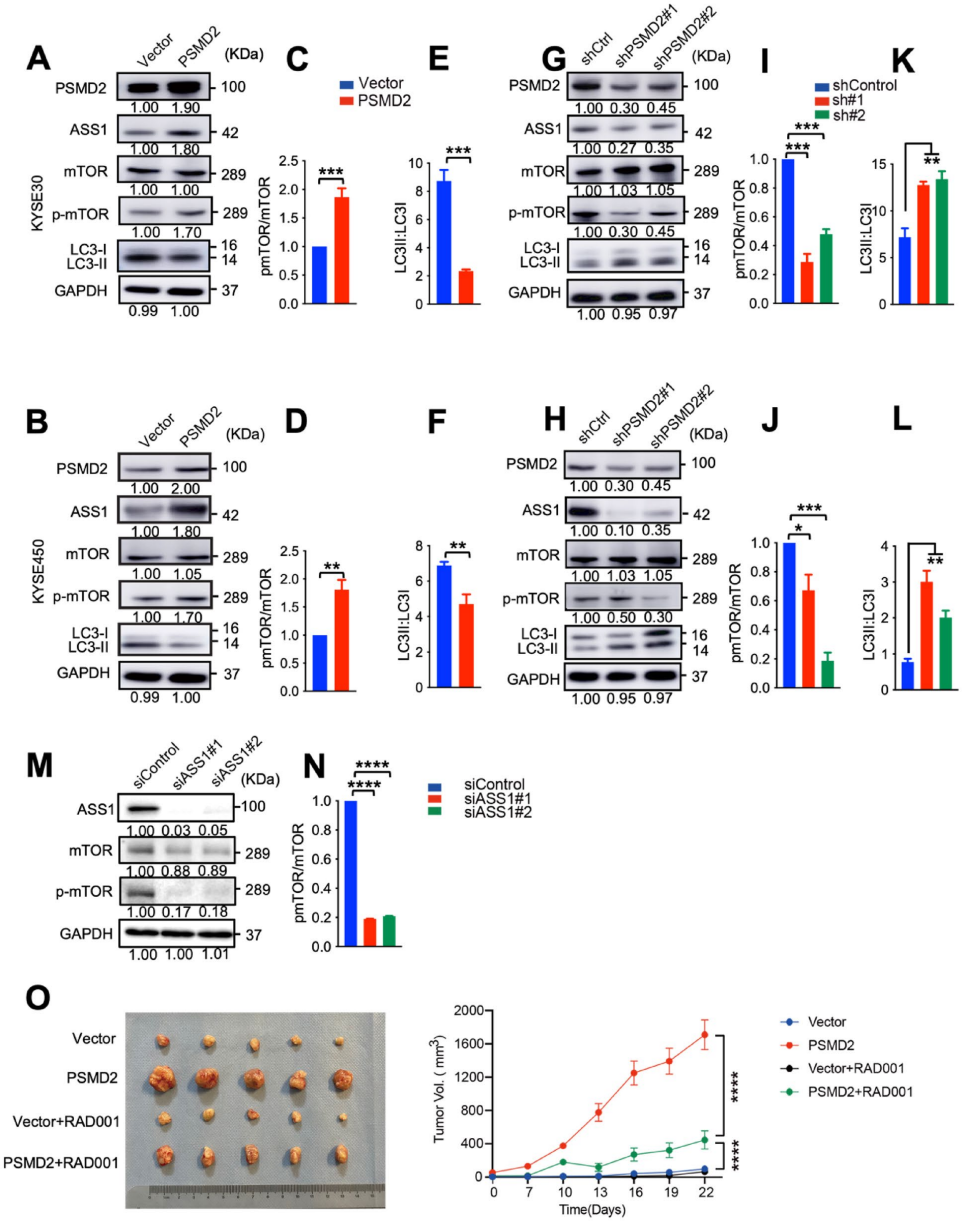
PSMD2 inhibits autophagy via ASS1-mTOR pathway. **A**, **B** The expression of indicated proteins in KYSE30 cells (**A**) and KYSE450 cells (**B**) with or without *PSMD2* overexpression. **C**, **D** The p-mTOR:mTOR ratio was qualified with ImageJ software in KYSE30 cells (**C**) and KYSE 450 cells (**D**) with or without *PSMD2* overexpression. N = 3. Data were presented as mean ± SD.**E**, **F** The LC3-II: LC3-I ratio was qualified with ImageJ software in KYSE30 cells (**E**) and KYSE 450 cells (**F**) with or without *PSMD2* overexpression. N = 3. Data were presented as mean ± SD.**G**, **H** The expression of the indicated proteins in KYSE30 cells (**G**) and KYSE450 cells (**H**) *PSMD2* knockdown in. **I**, **J** The pmTOR:mTOR total ratio was qualified with ImageJ software in KYSE30 cells (I) and KYSE 450 cells (**J**).**K**, **L** The LC3-II: LC3-I ratio was qualified with ImageJ software in KYSE30 cells (K) and KYSE 450 cells (**L**). N = 3. Data were presented as mean ± SD.**M**, **N** Western blot analysis of expression of the indicated molecules in *ASS1* knockdown in KYSE30 cells (M). The pmTOR:mTOR total ratio was qualified with ImageJ software. N = 3. Data were presented as mean ± SD.**O** RAD001 treatment significantly repressed the proliferation of *PSMD2* overexpressing KYSE450 cells implanted in mouse armpit (N = 5). Data were presented as mean ± SD, ****p < 0.0001

Because ASS1 is known to activate mTOR that inhibits autophagy [47], we examined the roles of PSMD2 on the expression and activation of mTOR in ESCC cells. Increased expression of *PSMD2* induced the phosphorylation levels of mTOR (Fig. 6A–D) while decreased the ratio of LC3II to LC3I (Fig. 6E, F). The silencing of *PSMD2* had the opposite effects (Fig. 6G–L). The knockdown of ASS1 decreased the phosphorylation levels of mTOR (Fig. 6M, N). These findings indicate that PSMD2-ASS1 pathway activates mTOR.

Everolimus (RAD001) is an mTOR inhibitor that also induces autophagy. When mice were treated with a placebo, tumors derived from *PSMD2* overexpressing cells grew at significantly faster rates compared with tumors derived from control cells. When ESCC bearing mice were treated with RAD001, the increased growth of tumors formed by *PSMD2* overexpressing ESCC cells was significantly suppressed by RAD001 treatment, indicating that mTOR mediates the roles of PSMD2 in promoting tumorigenesis of ESCC (Fig. 6O). Together, these findings support the notion that PSMD2-ASS1-mTOR pathway promotes ESCC proliferation by inhibiting autophagy.

## Discussion

PSMD2 is a newly identified receptor in the 19S regulatory particle of the proteasome, and its aberrant expression has been correlated with the progression of some types of cancer [13–16]. In this study, we found that the overexpression of PSMD2 is correlated with tumor aggression and poor survival of patients with ESCC. In addition, we revealed for the first time that PSMD2 may act as an autophagy inhibitor by inducing the expression of ASS1-mTOR pathway.

It has been known that autophagy can inhibit tumorigenesis by maintaining genome stability and inhibiting the accumulation of oncoproteins in cells [29–31]. In human breast and ovarian cancers, allelic deletion of the autophagy gene *BECN1* has been documented [32]. In addition, mice deficient in the essential autophagy genes such as *Atg4c*, *Atg5,* and *Atg7* are susceptible to tumorigenesis [33–35]. Oncogene activation may cause cancer and inflammation and the inflammatory conditions increase cancer risk. It has been shown that autophagy-deficient tumor tissues display an increased level of inflammation [36], indicating that the intact autophagy inhibits inflammation and cancer. Our findings that the depletion of ATG7 abrogates the inhibitory effects of *PSMD2* knockdown on ESCC cell proliferation indicate that the tumor-promoting roles of PSMD2 in ESCC depends on its activity to inhibit autophagy. However, the role of autophagy in cancer development is complex; other studies have suggested that autophagy has tumor-promoting effects [37–40]. Therefore, a better understanding of the context-specific role of autophagy in certain cancers and the mechanism involved will be crucial for effective autophagy-based cancer therapy [41, 42].

Using DIA proteomics analysis, we demonstrate that overexpression of PSMD2 increases the expression levels of ASS1, a key enzyme in the urea cycle [43]. In the liver, ASS1 produces argininosuccinate from citrulline and aspartate. Outside the liver, ASS1 and its subsequent urea cycle enzyme argininosuccinate lyase regulate the citrulline-arginine cycle, both of which supply arginine and its downstream metabolites, including polyamines, nitric oxide, and proline, in a cell-specific manner [44, 45]. Because autophagy may result from the deficiency of amino acids in cells [46, 47], the enhanced urea cycle caused by PSMD2-ASS1 overexpression, which provides ESCC cells with more amino acids, would be expected to inhibit autophagy and promote cell proliferation. Furthermore, consistent with previous findings that arginine activates mTOR [48], we have found that the mTOR pathway is activated upon PSMD2-ASS1 overexpression in ESCC cells, suggesting that PSMD2-ASS1 induced autophagy may be mediated by the mTOR pathway.

The identification of PSMD2 as an autophagy inhibitor in addition to being a ubiquitin receptor in the UPS system has potential clinical significance. Dual functions of PSMD2 might explain why the sole inhibition of the UPS system or autophagy is not effective in cancer treatment but instead promotes tumor progression [1, 58]. Applications of proteasome or mTOR inhibitors such as rapamycin derivatives [49, 50] to enhance cancer cell autophagy could therefore be a promising treatment strategy for ESCC with *PSMD2* overexpression. It has been well documented that ASS1 expression is differentially regulated in different types of cancer. In many cancer types, ASS1 expression is epigenetically downregulated and the low ASS1 levels correlated with poor survival in patients [51, 52]. However, increased ASS1 levels have also been noted in some types of malignancy such as gastric, colorectal, and ovarian cancers [53–55]. In the present study, we have demonstrated that ASS1 is upregulated in ESCC as a consequence of PSMD2 overexpression. Upregulation of ASS1 may result in dysregulation of the metabolic urea cycle and thus decreases pyrimidine synthesis [56]. The increased pyrimidine level in cancer cells can cause mutational bias favoring pyrimidine, leading to high levels of hydrophobic immunogenic antigens and hence increased response to immunotherapy [57, 58]. In the present study, we have found that more than 70% of ESCC samples overexpressed ASS1 compared with their adjacent normal tissues, suggesting that these tumors may respond poorly to immunotherapy. Therefore, a combination of autophagy activators and immune checkpoint inhibitors might be beneficial for the treatment of ESCC.

## Conclusions

In summary, we have identified PSMD2 as an oncogenic protein in ESCC and demonstrated their effects on autophagy inhibition via activating the ASS1-mTOR pathway, which significantly promotes ESCC cell proliferation in vitro and ESCC growth in vivo. These findings reveal a novel oncogenic role of PSMD2 in ESCC and provide a potential strategy for the treatment of PSMD2-overexpressing ESCC with autophagy activators.

## Supplementary Information

**Additional file 1: Fig. S1.** PSMD2 inhibits autophagy and promotes proliferation of KYSE450 cells. (A, B) PSMD2 overexpression reduced the formation of LC3B puncta in KYSE450 cells (A). PSMD2 knockdown increased the formation of LC3B puncta in KYSE450 cells (B). Autophagy of KYSE450 cells with PSMD2 overexpression or knockdown was induced by FBS deprivation for 3 h or 20 h. The cells were then stained with the DAPGreen (green). The nucleus was stained with Hoechst 33342 (blue). Scale bar: 30 μm. (C) The impact of ATG7 knockdown on the proliferation of ESCC cells with PSMD2 depletion. The cells were fluorescently stained with EdU (red). The nucleus was stained with DAPI (blue). The efficiency of ATG7 knockdown was shown in the upper right panel. The percentage of EdU-positive cells was shown in the bottom right panel. N=3. The data were presented as mean ± SD. Scale bar: 100 μm. (D) The impact of ATG7 knockdown on the colony formation of ESCC cells with PSMD2 depletion. The cells were seeded into 6-well plates with a density of 1000 cells per well. After being cultured for 14 days, the cells were stained with crystal violet. N=3. The data were presented as mean ± SD. (E) The impact of ATG7 knockdown on cell proliferation of ESCC cells with PSMD2 depletion. Cell numbers were determined by CCK-8 assay at the indicated time points. N=3. The data were presented as mean ± SD. * p < 0.05, ** p < 0.01, *** p < 0.001 and **** p < 0.0001. **Fig. S2.** PSMD2 knockdown induce autophagic fluxes. (A, B) The autophagic fluxes of KYSE30 cells (A) and KYSE450 cells (B) with PSMD2 knockdown. The cells were stably transduced to express mRFP-GFP-LC3 fusion protein. Scale bar: 10 μm. N=10. The data were presented as mean ± SD, *** p < 0.001 and **** p < 0.0001. **Fig. S3.** PSMD2 promotes the proliferation of ESCC cells. (A, B) Representative images of the xenograft tumors formed in NOD/scid mice inoculated with KYSE30 cells (A) and KYSE450 cells (B) with or without PSMD2 knockdown. Growth curves of tumors derived from the indicated cell lines were shown. (C) PSMD2 knockdown significantly inhibited the migration and invasion capabilities of ESCC cells. The left panels were representative images of ESCC cells in transwell assays. The right panels were statistical data. N=3. Data were presented as mean ± SD. *p< 0.05, **p < 0.01, ***p< 0.001, and ****p < 0.0001. **Fig. S4.** ASS1 mediates the PSMD2-dependent inhibition of autophagy. (A) Representative H&E and IHC images of tumors formed by KYSE450 cells with PSMD2 overexpression. The expression levels of PSMD2, ASS1, LC3 and p62 are shown. Right panel, quantitative analyses of the positive cell numbers. N=5. Data were presented as mean ± SD. (B) Representative IHC images of p62 expression in tumors formed by KYSE30 cells with PSMD2 overexpression or knockdown. (C) Quantitative analyses of the expression of PSMD2, ASS1, LC3 and p62 in tumors formed by KYSE30 cells with PSMD2 overexpression. N=5. Data were presented as mean ± SD. *p< 0.05, **p < 0.01, ***p< 0.001, and ****p < 0.0001. (D) Quantitative analyses of the expression of PSMD2, ASS1, LC3 and p62 in tumors formed by KYSE30 cells with PSMD2 knockdown. N=5. Data were presented as mean ± SD. *p< 0.05, **p < 0.01, ***p< 0.001, and ****p < 0.0001. **Fig. S5.** ASS1 mediates the roles of PSMD2 in the progression of ESCC. (A) The relationship between PSMD2 and ASS1 protein levels in ESCC determined by IHC staining. Showing representative IHC images of PSMD2 and ASS1 expression in serial sections of ESCC tissue array (n = 144). Scale bar in left images = 600 μm. Scale bar in right images = 100 μm. (B, C) Effect of ASS1 knockdown on cell proliferation in KYSE30 cells (B) and KYSE450 cells (C) after the change of PSMD2 expression. The cell proliferation rate was determined by CCK-8 assay. The cells were seeded into 6-well plate at a density of 3000 cells per well for KYSE30 cells or 1000 cells per well for KYSE450 cells. After being cultured for 14 days, the cells were then stained with crystal violet. Quantification of the data is shown. Data were presented as mean ± SD of three independent experiments, **p < 0.01, ***p < 0.001, and ****p < 0.0001. **Fig. S6.** The effects of ASS1 on ESCC cell proliferation caused by the change of PSMD2 expression levels. (A) Effect of ASS1 knockdown on colony formation in KYSE30 cells (upper panel) and KYSE450 cells (bottom panel) with PSMD2 overexpression. (B) Effect of ASS1 overexpression on colony formation in KYSE30 cells (upper panel) and KYSE450 cells (bottom panel) with PSMD2 depletion. Data were presented as mean ± SD of three independent experiments, **p <0.01, ***p <0.001, and ****p <0.0001. Table S1. Sequences of siRNAs and shRNAs used in this study. Table S2. 26S proteasome gene expression with paried t-test and survival with logrank test in esophageal squamous cell carcinoma data. Table S3. Protein level change in KYSE30 cells with PSMD2 knockdown related to the corresponding control.

**Additional file 2: Table S4.** Characteristics of 144 patients with esophageal squamous-cell carcinoma in this study

## Data Availability

Data and materials will be shared.

## Abbreviations

ATG7: Autophagy-related 7
CQ: Chloroquine diphosphate salt
ESCC: Esophageal squamous cell carcinoma
ASS1: Argininosuccinate synthase 1
FBS: Fetal bovine serum
IHC: Immunohistochemical
KEGG: Kyoto Encyclopedia of Genes and Genomes
LC3B: Light chain 3B
mTORC1: Mammalian target of rapamycin complex 1
RP: Regulatory particle
UPS: Ubiquitin–proteasome system
