# Supplementary material for: PSMD2 contributes to the progression of esophageal squamous cell carcinoma by repressing autophagy
Source: Cell Biosci. 2023 Mar 30;13:67. doi: 10.1186/s13578-023-01016-4 (PMC10061715; doi:10.1186/s13578-023-01016-4)
Supplement: Supplementary file 1 — Additional file 1: Fig. S1. PSMD2 inhibits autophagy and promotes proliferation of KYSE450 cells. (A, B) PSMD2 overexpression reduced the formation of LC3B puncta in KYSE450 cells (A). PSMD2 knockdown increased the formation of LC3B puncta in KYSE450 cells (B). Autophagy of KYSE450 cells with PSMD2 overexpression or knockdown was induced by FBS deprivation for 3 h or 20 h. The cells were then stained with the DAPGreen (green). The nucleus was stained with Hoechst 33342 (blue). Scale bar: 30 μm. (C) The impact of ATG7 knockdown on the proliferation of ESCC cells with PSMD2 depletion. The cells were fluorescently stained with EdU (red). The nucleus was stained with DAPI (blue). The efficiency of ATG7 knockdown was shown in the upper right panel. The percentage of EdU-positive cells was shown in the bottom right panel. N=3. The data were presented as mean ± SD. Scale bar: 100 μm. (D) The impact of ATG7 knockdown on the colony formation of ESCC cells with PSMD2 depletion. The cells were seeded into 6-well plates with a density of 1000 cells per well. After being cultured for 14 days, the cells were stained with crystal violet. N=3. The data were presented as mean ± SD. (E) The impact of ATG7 knockdown on cell proliferation of ESCC cells with PSMD2 depletion. Cell numbers were determined by CCK-8 assay at the indicated time points. N=3. The data were presented as mean ± SD. * p < 0.05, ** p < 0.01, *** p < 0.001 and **** p < 0.0001. Fig. S2. PSMD2 knockdown induce autophagic fluxes. (A, B) The autophagic fluxes of KYSE30 cells (A) and KYSE450 cells (B) with PSMD2 knockdown. The cells were stably transduced to express mRFP-GFP-LC3 fusion protein. Scale bar: 10 μm. N=10. The data were presented as mean ± SD, *** p < 0.001 and **** p < 0.0001. Fig. S3. PSMD2 promotes the proliferation of ESCC cells. (A, B) Representative images of the xenograft tumors formed in NOD/scid mice inoculated with KYSE30 cells (A) and KYSE450 cells (B) with or without PSMD2 knockdown. Growth curve [file 13578_2023_1016_MOESM1_ESM.pdf]

Additional file

Supplementary materials

Liu et al. Supplementary Figure1

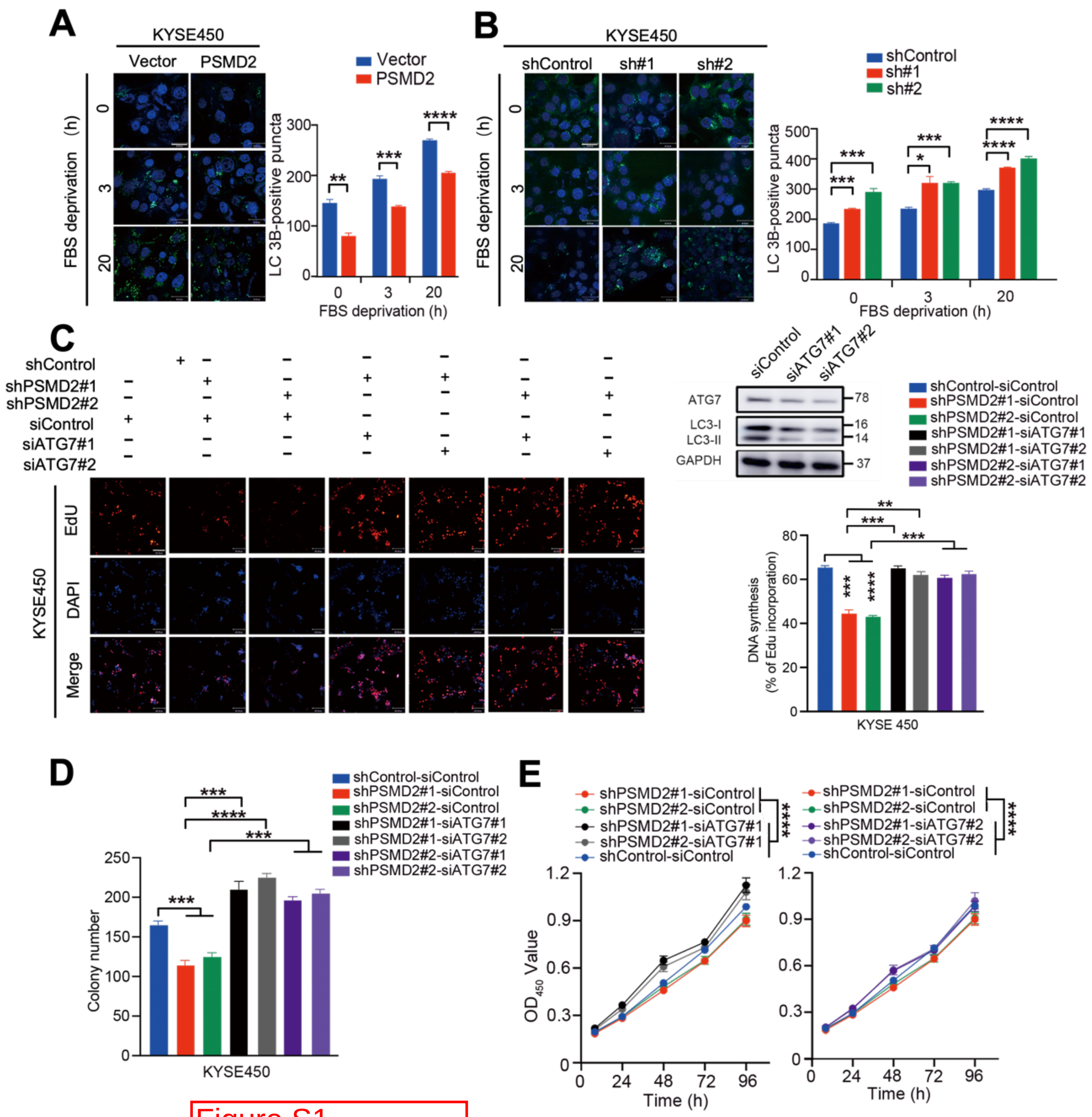

Figure S1

Supplementary Figure 1. PSMD2 inhibits autophagy and promotes proliferation of KYSE450 cells.

(**A**, **B**) *PSMD2* overexpression reduced the formation of LC3B puncta in KYSE450 cells (A). *PSMD2* knockdown increased the formation of LC3B puncta in KYSE450 cells (B). Autophagy of KYSE450 cells with *PSMD2* overexpression or knockdown was induced by FBS deprivation for 3 h or 20 h. The cells were then stained with the DAPGreen (green). The nucleus was stained with Hoechst 33342 (blue). Scale bar: 30 µm.

(**C**) The impact of *ATG7* knockdown on the proliferation of ESCC cells with *PSMD2* depletion. The cells were fluorescently stained with EdU (red). The nucleus was stained with DAPI (blue). The efficiency of ATG7 knockdown was shown in the upper right

panel. The percentage of EdU-positive cells was shown in the bottom right panel. N=3. The data were presented as mean ± SD. Scale bar: 100 μm.

(**D**) The impact of *ATG7* knockdown on the colony formation of ESCC cells with *PSMD2* depletion. The cells were seeded into 6-well plates with a density of 1000 cells per well. After being cultured for 14 days, the cells were stained with crystal violet. N=3. The data were presented as mean ± SD.

(**E**) The impact of *ATG7* knockdown on cell proliferation of ESCC cells with *PSMD2* depletion. Cell numbers were determined by CCK-8 assay at the indicated time points. N=3. The data were presented as mean ± SD. * $p < 0.05$, ** $p < 0.01$, *** $p < 0.001$ and **** $p < 0.0001$.

Liu et al. Supplementary Figure2

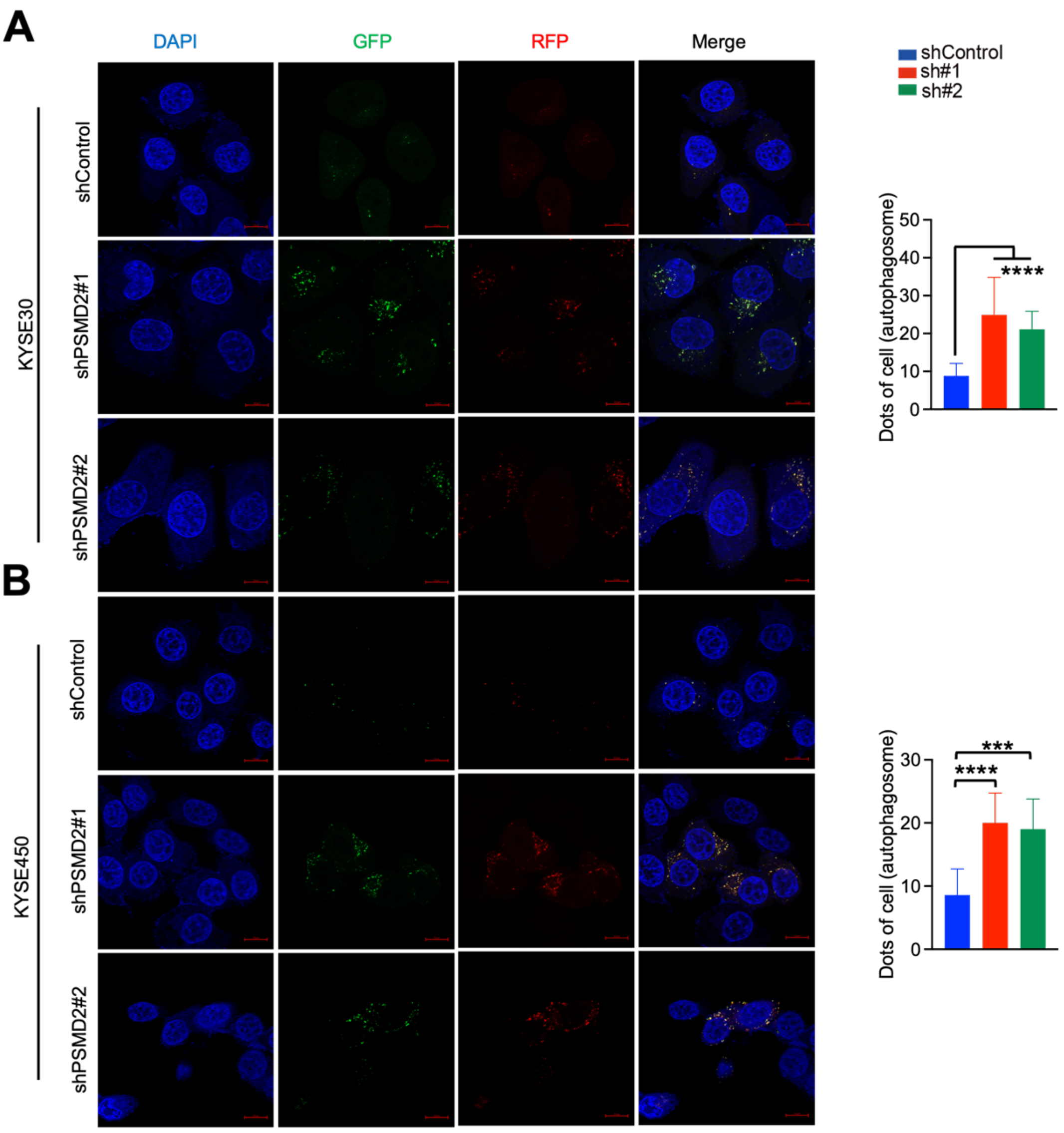

Figure S2

Supplementary Figure 2. PSMD2 knockdown induce autophagic fluxes. (**A, B**) The autophagic fluxes of KYSE30 cells (A) and KYSE450 cells (B) with *PSMD2* knockdown. The cells were stably transduced to express mRFP-GFP-LC3 fusion protein. Scale bar: 10 µm. N=10. The data were presented as mean ± SD, *** $p < 0.001$ and **** $p < 0.0001$.

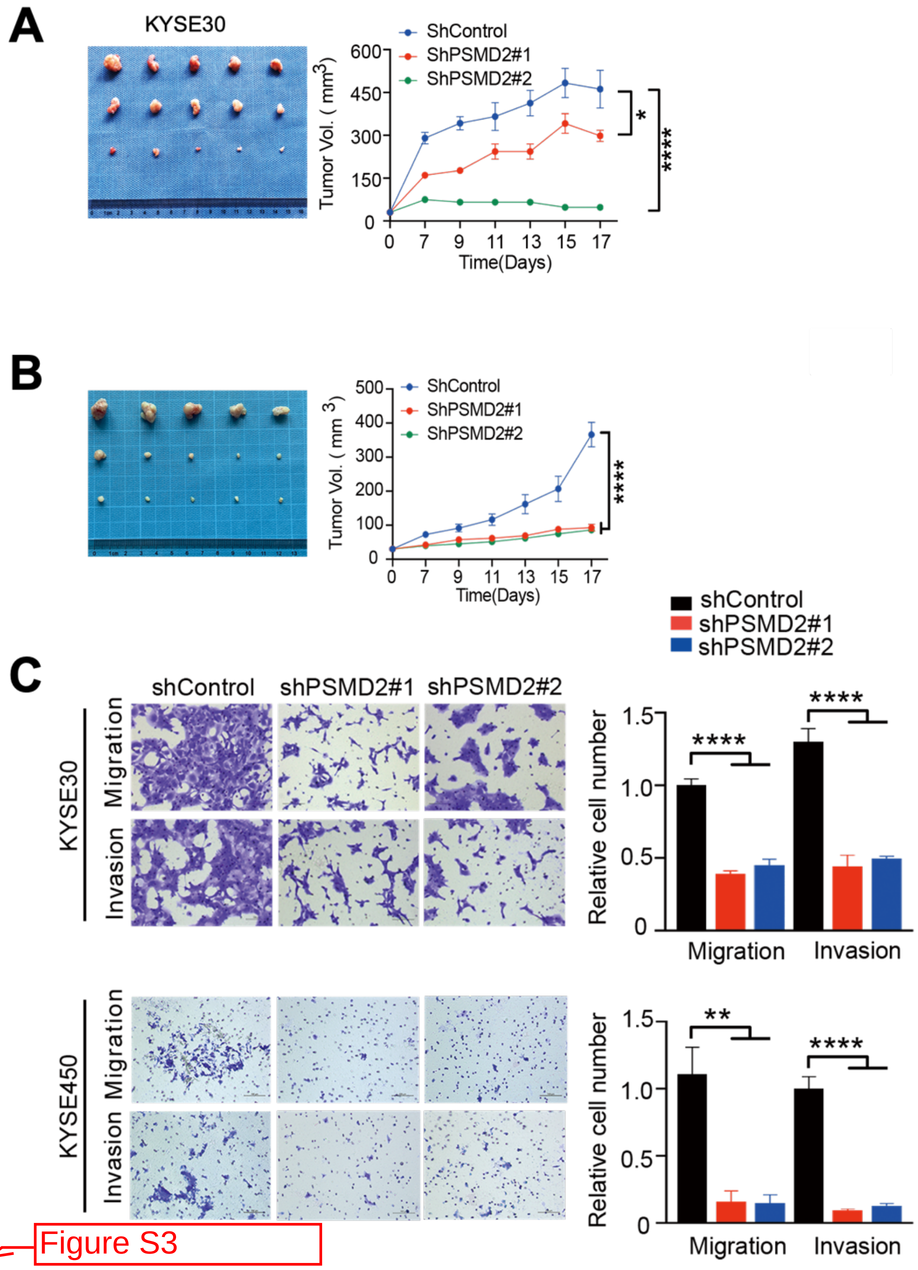

Figure S3

Supplementary Figure 3. PSMD2 promotes the proliferation of ESCC cells. (**A**, **B**) Representative images of the xenograft tumors formed in NOD/*scid* mice inoculated with KYSE30 cells (A) and KYSE450 cells (B) with or without *PSMD2* knockdown. Growth curves of tumors derived from the indicated cell lines were shown. (**C**) *PSMD2* knockdown significantly inhibited the migration and invasion capabilities of ESCC cells. The left panels were representative images of ESCC cells in transwell assays. The right panels were statistical data. N=3. Data were presented as mean ± SD. *$p < 0.05$, **$p < 0.01$, ***$p < 0.001$, and ****$p < 0.0001$.

Liu et al. Supplementary Figure4

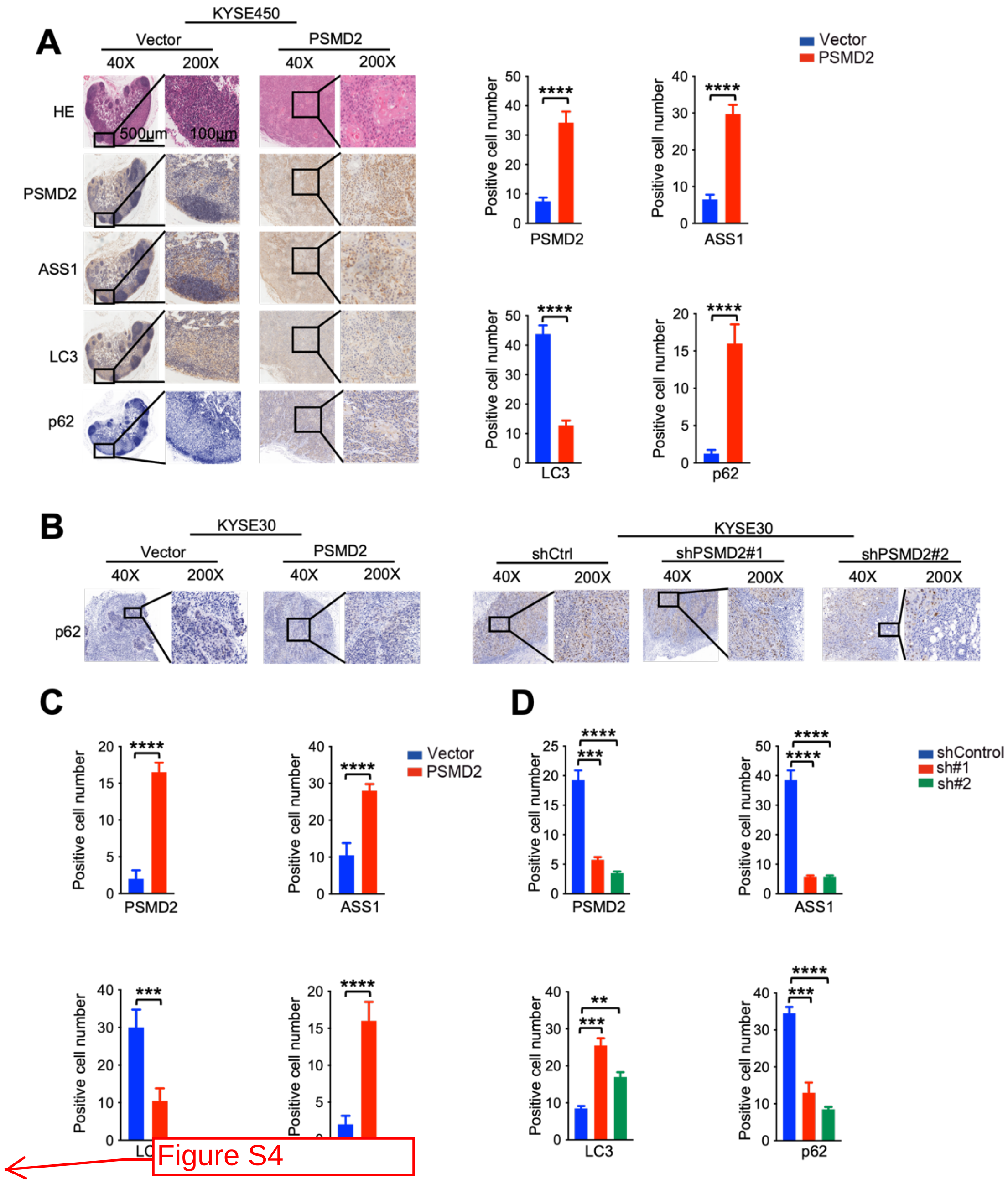

Supplementary Figure 4. ASS1 mediates the PSMD2-dependent inhibition of autophagy.

(**A**) Representative H&E and IHC images of tumors formed by KYSE450 cells with *PSMD2* overexpression. The expression levels of PSMD2, ASS1, LC3 and p62 are shown. Right panel, quantitative analyses of the positive cell numbers. N=5. Data were presented as mean ± SD.

(B) Representative IHC images of p62 expression in tumors formed by KYSE30 cells with *PSMD2* overexpression or knockdown.

(**C**) Quantitative analyses of the expression of PSMD2, ASS1, LC3 and p62 in tumors formed by KYSE30 cells with *PSMD2* overexpression. N=5. Data were presented as mean ± SD. **p*< 0.05, ***p* < 0.01, ****p*< 0.001, and *****p* < 0.0001.

(**D**) Quantitative analyses of the expression of PSMD2, ASS1, LC3 and p62 in tumors

formed by KYSE30 cells with *PSMD2* knockdown. N=5. Data were presented as mean ± SD. *$p < 0.05$, **$p < 0.01$, ***$p < 0.001$, and ****$p < 0.0001$.

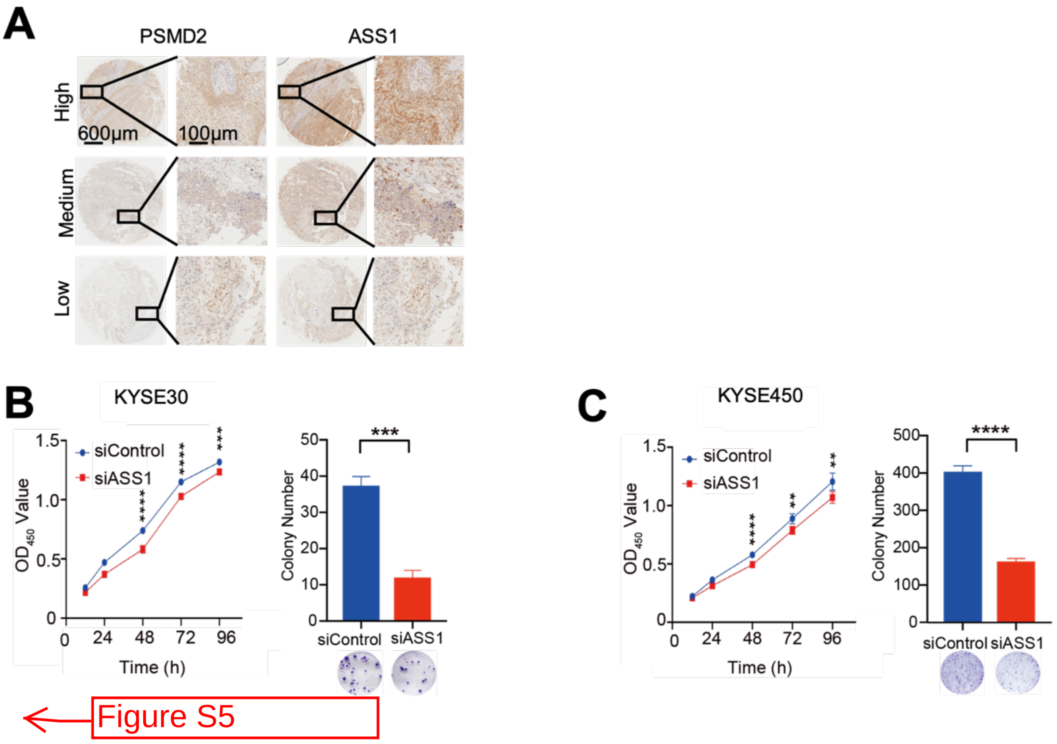

Figure S5

Supplementary Figure 5. ASS1 mediates the roles of PSMD2 in the progression of ESCC.

(**A**) The relationship between PSMD2 and ASS1 protein levels in ESCC determined by IHC staining. Showing representative IHC images of PSMD2 and ASS1 expression in serial sections of ESCC tissue array (n = 144). Scale bar in left images = 600 μm. Scale bar in right images = 100 μm.

(**B**, **C**) Effect of ASS1 knockdown on cell proliferation in KYSE30 cells (B) and KYSE450 cells (C) after the change of PSMD2 expression. The cell proliferation rate was determined by CCK-8 assay. The cells were seeded into 6-well plate at a density of 3000 cells per well for KYSE30 cells or 1000 cells per well for KYSE450 cells. After being cultured for 14 days, the cells were then stained with crystal violet. Quantification of the data is shown. Data were presented as mean ± SD of three independent experiments, $^{**}p < 0.01$, $^{***}p < 0.001$, and $^{****}p < 0.0001$.

Liu et al. Supplementary Figure6

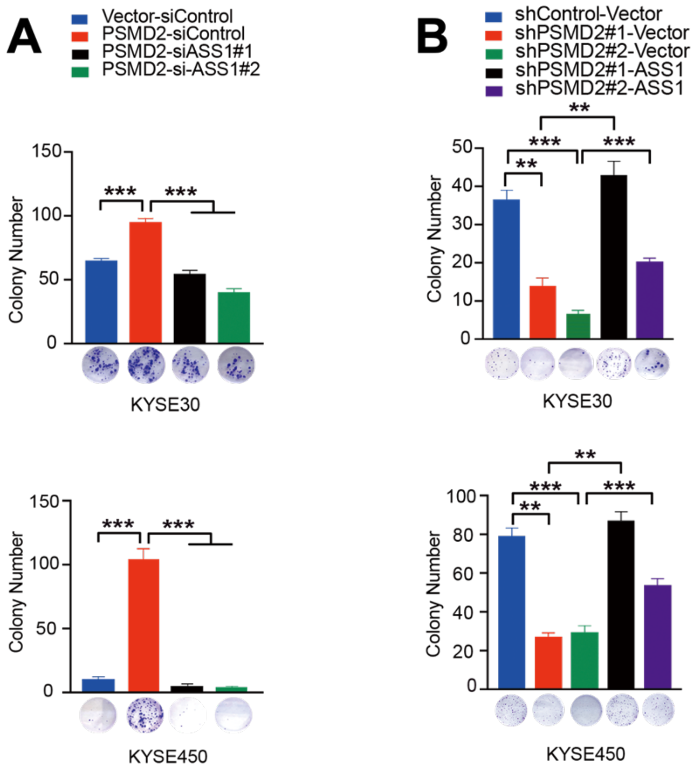

Figure S6

Supplementary Figure 6. The effects of ASS1 on ESCC cell proliferation caused by the change of PSMD2 expression levels.

(**A**) Effect of ASS1 knockdown on colony formation in KYSE30 cells (upper panel) and KYSE450 cells (bottom panel) with PSMD2 overexpression.

(**B**) Effect of ASS1 overexpression on colony formation in KYSE30 cells (upper panel) and KYSE450 cells (bottom panel) with PSMD2 depletion. Data were presented as mean ± SD of three independent experiments, $**p<0.01$, $***p<0.001$, and $****p<0.0001$.

Table S1. Sequences of siRNAs and shRNAs used in this study.

Primer	Used for	Sequence (5'-3')
shPSMD2#1	Knockdown	GCTGGCTCAAATCGTGAAGAT
		ATCTTCACGATTTGAGCCAGC
shPSMD2#2	Knockdown	CCACATTTGTAGCGAACACTT
		AAGTGTTCGCTACAAATGTGG
siPSMD2#1	Knockdown	CCACUAUCCUUCAGACCAUTT
		AUGGUCUGAAGGAUAGUGGTT
siPSMD2#2	Knockdown	GGUGGAUGUGUGUGCAUAUTT
		AUAUGCACACACAUCCACCTT
siASS1#1	Knockdown	GAACAAGGCUAUGACGUCATT
		UGACGUCAUAGCCUUGUUCTT
siASS1#2	Knockdown	CUGACAUUCUCGAGAUCGATT
		UCGAUCUCGAGAAUGUCAGTT
siAtg7#1	Knockdown	GAAGCUCCCAAGGACAUUATT
		UAAUGUCCUUGGGAGCUUCTT
siAtg7#2	Knockdown	GCCGUGGAAUUGAUGGUAUTT
		AUACCAUCAAUUCCACGGCTT

Table S2. 26S proteasome gene expression with paried t-test and survival with log-rank test in esophageal squamous cellcarcinoma data.

		Paired_t_test			log_rank	
genes	ID	*P*	FDR	FC	*P*_value （median）	*P*_value （mean）
PSMD2	5708	1.04E-33	4.58E-32	2.05	0.04	0.04
PSMA7	5688	2.74E-30	6.76E-29	1.83	0.22	0.14
ADRM1	11047	2.49E-29	5.24E-28	1.73	0.42	0.58
PSMB4	5692	3.31E-29	6.87E-28	1.67	0.08	0.27
PSMB5	5693	7.79E-28	1.29E-26	1.77	0.10	0.08
PSMA6	5687	4.98E-26	6.26E-25	2.13	0.38	0.49
PSMD11	5717	2.27E-25	2.62E-24	1.75	0.01	0.04
PSMA2	5683	9.5E-25	1.02E-23	1.59	0.40	0.46
PSMB1	5689	7.87E-21	5.39E-20	1.50	0.05	0.05
PSMD9	5715	8.14E-21	5.56E-20	1.43	0.90	0.75
PSMB9	5698	1.71E-20	1.13E-19	2.88	0.01	0.01
PSMB7	5695	5.19E-19	2.95E-18	1.54	0.19	0.23
PSMB10	5699	1.08E-17	5.46E-17	1.83	0.02	0.02
PSMC1	5700	2.54E-16	1.13E-15	1.48	0.55	0.24
PSME4	23198	2.73E-16	1.21E-15	1.46	0.04	0.07
PSMD4	5710	1.17E-15	4.9E-15	1.35	0.03	0.02
PSMD7	5713	4.24E-15	1.69E-14	1.34	0.47	0.23
PSMD13	5719	5.69E-15	2.24E-14	1.37	0.58	0.95
PSMB6	5694	1.78E-12	5.71E-12	1.30	0.94	0.94

Table S3. Protein level change in KYSE30 cells with PSMD2 knockdown related to the corresponding control.

Protein	Gene	Log2FC	*P* value
AGRV1_HUMAN	ADGRV1	-4.16	2.10E-03
K1C17_HUMAN	KRT17	-3.45	6.38E-06
GTR3_HUMAN	SLC2A3	-3.32	3.38E-03
PXL2A_HUMAN	PRXL2A	-2.81	1.92E-03
GPSM1_HUMAN	GPSM1	-2.80	1.80E-03
ID3_HUMAN	ID3	-2.34	5.59E-04
SUSD2_HUMAN	SUSD2	-2.31	3.70E-04
KCRB_HUMAN	CKB	-2.22	1.36E-04
SNG1_HUMAN	SYNGR1	-2.19	2.67E-03
CLD1_HUMAN	CLDN1	-2.03	7.41E-05
ASSY_HUMAN	ASS1	-1.94	2.32E-04
MACD1_HUMAN	MACROD1	-1.94	4.61E-08
K2C6B_HUMAN	KRT6B	-1.91	8.60E-03
S27A1_HUMAN	SLC27A1	-1.87	7.64E-04
ALBU_HUMAN	ALB	-1.86	7.06E-03
SCRN2_HUMAN	SCRN2	-1.85	7.27E-03
ANK3_HUMAN	ANK3	-1.84	8.19E-04
PTPRF_HUMAN	PTPRF	-1.82	1.40E-04
ARH40_HUMAN	ARHGEF40	-1.69	3.67E-02
SIR3_HUMAN	SIRT3	-1.66	2.15E-06
IDHP_HUMAN	IDH2	-1.61	6.05E-05
CLIC3_HUMAN	CLIC3	-1.60	1.15E-05
SIDT2_HUMAN	SIDT2	-1.60	1.21E-02
LG3BP_HUMAN	LGALS3BP	-1.56	2.13E-03
DHCR7_HUMAN	DHCR7	-1.44	1.54E-07
F210A_HUMAN	FAM210A	-1.42	1.25E-02
AHDC1_HUMAN	AHDC1	-1.41	3.29E-02
MMSA_HUMAN	ALDH6A1	-1.39	7.62E-03
GLNA_HUMAN	GLUL	-1.38	3.43E-02
PTPRD_HUMAN	PTPRD	-1.36	3.75E-04
ARRB1_HUMAN	ARRB1	-1.36	1.29E-03
SDHF2_HUMAN	SDHAF2	-1.35	1.30E-03
K1C14_HUMAN	KRT14	-1.35	8.11E-04
PSMD2_HUMAN	PSMD2	-1.31	3.19E-07
DSG3_HUMAN	DSG3	-1.30	1.39E-03
HYI_HUMAN	HYI	-1.25	6.20E-03
KLHL9_HUMAN	KLHL9	-1.25	1.64E-07

Table S4. Characteristics of 144 patients with esophageal squamous-cell carcinoma in this study (Excel version)
